# Sources of bias in COVID-19 infection fatality rate estimation: Angelo Maria Pezzullo

**DOI:** 10.1093/eurpub/ckac129.128

**Published:** 2022-10-25

**Authors:** AM Pezzullo

**Affiliations:** 1 Dipartimento di Sanità Pubblica, Università Cattolica del Sacro Cuore, Rome, Italy; 2 Meta-Research Innovation Center at Stanford, Stanford University, Stanford, USA

## Abstract

Since the beginning of the devastating COVID-19 pandemic, there has been a great debate about various public health relevant parameters such as the number of people infected with SARS-CoV-2, the number of deaths from COVID-19, and the resulting infection fatality rate (IFR), calculated as a ratio of the number of deaths from COVID-19 and the number of people infected with SARS-CoV-2. Among people dying from COVID-19, the largest burden is carried by the elderly, and locations with an older population will have a higher average IFR. Drawing from a project on the estimation of age-stratified IFR at an international level, in this methodological presentation, I will review the considered sources of bias for COVID-19 IFR calculation and interpretation. Both numerator and denominator can be overestimated or underestimated leading to biased estimates, while different locations can present sources of true variability. The estimation of the number of people infected with SARS-CoV-2 (the denominator of the IFR) presents several challenges. Relying on testing is inadequate due to a substantial undiagnosed proportion, and seroprevalence studies have been used to estimate the number of people infected with COVID-19, but selection bias can arise when the examined population might have a lower or higher risk than the target population. This can be the case when factors such as ethnicity, working status, and comorbidity are not considered in the recruitment. Information bias can result from suboptimal test performance and seroreversion. The number of deaths can be underestimated in situations where testing is not widely available and overestimated by the attribution of COVID-19 deaths to patients that have died with COVID-19 but not from it. Sources of true variability between locations are the population age distribution, protection of the vulnerable populations, as well as the presence of a prepared and efficient healthcare system.

